# Hebbian plasticity requires compensatory processes on multiple timescales

**DOI:** 10.1098/rstb.2016.0259

**Published:** 2017-03-05

**Authors:** Friedemann Zenke, Wulfram Gerstner

**Affiliations:** 1 Department of Applied Physics, Stanford University, Stanford, CA 94305, USA; 2 Brain Mind Institute, School of Life Sciences and School of Computer and Communication Sciences, Ecole Polytechnique Fédérale de Lausanne, 1015 Lausanne EPFL, Switzerland

**Keywords:** Hebbian plasticity, homeostasis, rapid compensatory processes, heterosynaptic plasticity, synaptic scaling, metaplasticity

## Abstract

We review a body of theoretical and experimental research on Hebbian and homeostatic plasticity, starting from a puzzling observation: while homeostasis of synapses found in experiments is a slow compensatory process, most mathematical models of synaptic plasticity use rapid compensatory processes (RCPs). Even worse, with the slow homeostatic plasticity reported in experiments, simulations of existing plasticity models cannot maintain network stability unless further control mechanisms are implemented. To solve this paradox, we suggest that in addition to slow forms of homeostatic plasticity there are RCPs which stabilize synaptic plasticity on short timescales. These rapid processes may include heterosynaptic depression triggered by episodes of high postsynaptic firing rate. While slower forms of homeostatic plasticity are not sufficient to stabilize Hebbian plasticity, they are important for fine-tuning neural circuits. Taken together we suggest that learning and memory rely on an intricate interplay of diverse plasticity mechanisms on different timescales which jointly ensure stability and plasticity of neural circuits.

This article is part of the themed issue ‘Integrating Hebbian and homeostatic plasticity’.

## 1. Introduction

Homeostasis refers to a family of compensatory processes at different spatial and temporal scales whose objective is to maintain the body, its organs, the brain or even individual neurons in the brain in a dynamic regime where they function optimally. A well-known example is the homeostatic regulation of body temperature in mammals, maintained at about 37°C independently of weather condition and air temperature. In neuroscience, homeostasis or homeostatic plasticity often refers to a compensatory process that stabilizes neural firing rates. In a classic experiment, cultured neurons that normally fire at, say 5 Hz, change their firing rate after a modulation of the chemical conditions in the culture, but eventually return to their target rate of 5 Hz during the following 24 h [1]. Thus, the experimentally best-studied form of homeostasis acts on a timescale of hours to days. This slow form of homeostatic plasticity manifests itself as the rescaling of the efficacy or weight of all afferent synapses onto a single neuron by a fixed fraction, for instance 0.78. This phenomenon is called ‘synaptic scaling’ [1].

Mathematical models of neural networks often make use of compensatory processes similar to synaptic scaling to stabilize firing rates in the presence of Hebbian plasticity. Hebbian plasticity is a form of synaptic plasticity which is induced by and further amplifies correlations in neuronal activity. It has been observed in many brain areas and can be induced quickly on a timescale of seconds to minutes. Its effect, however, is often long-lasting. It can last hours, days and possibly a lifetime. Owing to these properties, Hebbian plasticity is widely assumed to be the neural basis of associative long-term memory [2–4]. Moreover, Hebbian learning is thought to be the basis of developmental changes such as receptive field development [5–9].

**Figure 1.**
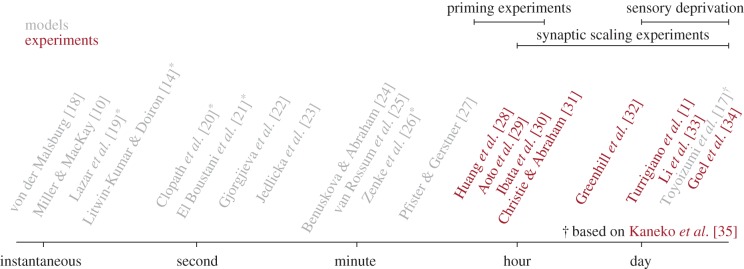
The timescales of synaptic scaling or metaplasticity are faster in models than reported in experiments. Here, we plot the timescale of either synaptic scaling or homeostatic metaplasticity as used in influential modeling studies (light grey). For comparison, we plot the typical readout time for experimental studies on synaptic scaling and metaplasticity (dark red). Publications suffixed with * describe network models as opposed to the other studies which relied on single neurons. Note that the model marked with † by Toyoizumi *et al*. [17] is an interesting case which has both RCPs and a slow form of homeostasis. Here, we have aligned it according to its homeostatic timescale. Work referenced in the figure: [10,14,17–35].

However, Hebbian plasticity alone leads to a positive feedback loop in which correlations of pre- and postsynaptic firing drive potentiation of synapses that increase postsynaptic rates and correlations further, which is unstable. To avoid pathological runaway dynamics of neural activity in mathematical models, it is necessary to add appropriate constraints to plasticity models [10,11]. A typical example of such a constraint is the normalization or rescaling of the sum of afferent synaptic weights: when the weight of one synaptic connection increases, weights of other connections onto the same neuron are algorithmically decreased to keep the total input constant or close to the optimal target regime. At a first glance, this form of multiplicative normalization [10] seems virtually identical to homeostatic ‘synaptic scaling’ introduced above. However, these two mechanisms are fundamentally distinct because they act on vastly different timescales. While normalization in models typically takes place on a timescale of seconds or less [10,12–14], in biology the effects of synaptic scaling manifest themselves only after hours [15,16]. A similar observation holds for homeostatic metaplasticity, which exists on timescales ranging from some tens of minutes to days (figure 1) [36,37]. Moreover, the difference between experimental data and models cannot be explained by a simple rescaling of time in the models, because the problem persists for quantitative plasticity models which capture the time course of biological data.

However, this difference in timescales may challenge the popular view that in biology Hebbian plasticity is constrained through homeostatic plasticity [16,38–41]. The algorithmic normalization of synaptic weights every second is not the same mechanism as the biological rescaling of synaptic weights over hours. Although, in the theoretical literature, a rapid stabilizing mechanism is typically called ‘homeostatic’, here we will refer to this class of control mechanisms as rapid compensatory processes (RCPs). The term ‘homeostatic plasticity’ is in the following reserved for slow negative feedback processes on the timescale of hours or days—a terminology that seems consistent with the available experimental literature [15,16]. In this review, we focus on this discrepancy of timescales and ask which biologically plausible processes could constrain Hebbian plasticity. Specifically, we will try to answer the following questions: Why do we need RCPs to stabilize Hebbian plasticity? How fast do these processes have to be—hours, minutes, seconds or less? Which mechanisms could fill this role in Hebbian learning? Moreover, what are the consequences of fast control mechanisms on memory formation and recall in network models? And finally, if RCPs are a requirement, what is the role of slower forms of negative feedback implemented by known forms of homeostatic plasticity?

## 2. Models of synaptic plasticity

Synaptic plasticity exists across different timescales. For instance, synaptic changes induced by a sequence of four presynaptic spikes in rapid sequence typically decay within a few hundred milliseconds [42–44] and are called short-term plasticity. The rapid decay implies that the changes are not useful for long-term memory formation, but more likely involved in gain control [42].

Other forms of plasticity induced by classic induction protocols [45–47] can have long-term effects on the timescale of hours or more. Long-term plasticity is therefore potentially useful for memory formation [2]. We remind the reader that the induction of long-term plasticity can be as fast as seconds, but the induced changes persist for much longer. Depending on the direction of synaptic change, we speak of long-term potentiation (LTP) or long-term depression (LTD).

Under suitable conditions the changes induced by a protocol of LTP or LTD are further consolidated after about an hour [48–50]. These effects are often referred to as late-phase long-term plasticity. In the rest of the paper, we focus on plasticity induction and the early phase of long-term plasticity and neglect consolidation and maintenance.

The diverse effects of long-term plasticity can be cast into a mathematical framework which describes the change of synaptic efficacy over time. Apart from a few notable exceptions [17,51–54], the vast majority of models of long-term plasticity assume a one-dimensional synaptic state space which represents the synaptic efficacy or weight *w_ij_* of a synapse from neuron *j* to neuron *i* [5,8,9,27,55–65]. The evolution *w_ij_* is then characterized by the differential equation2.1
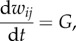
in which the function *G*, often called the ‘learning rule’, is a member of an infinite dimensional function space 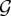, the space of all possible learning rules. This space is strongly constrained if we only focus on plausible learning rules, which are the rules in agreement with experimental findings.

For example, classical stimulation protocols for LTP [45–47], LTD [66,67] or spike-timing-dependent plasticity (STDP) [68–70], combine the activation of a presynaptic neuron, or a presynaptic pathway, with an activation, depolarization or chemical manipulation of the postsynaptic neurons, to induce synaptic changes. In models, this is typically formalized by stating that *G* only depends on quantities which are locally accessible to the synapse. It is customary to assume that the main locally accessible variables include: (i) the current synaptic state *w_ij_*; (ii) the activity pre*_j_* of the presynaptic neuron; and (iii) the state post*_i_* of the postsynaptic neuron [64,71,72]. Thus, we can write 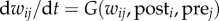. Additionally, *G* could also depend on low-dimensional information carried by chemical signals such as neuromodulators (see Frémaux & Gerstner [73] for a review).

Most published learning rules *G* can be written as the linear sum of different terms in which each term can be interpreted as a specific manifestation of plasticity. These terms act together to explain the measured outcome in plasticity experiments. Let us explain the most common ones using the following example learning rule:2.2
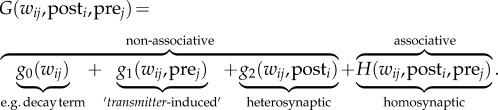


We will discuss each of the terms, going from right to left.

*H*: Co-stimulation of a presynaptic pathway (or presynaptic neuron) and a postsynaptic neuron, as used in many classic induction protocols of LTD or LTP, changes the activated synapses. Such input specific changes are called ‘homosynaptic’ because they affect *the same* synapses that are stimulated. In the above equation, the homosynaptic changes are characterized by the term *H* on the right, where *H* is shorthand for ‘Hebbian’. The homosynaptic changes are often further separable into individual contributions of LTD and LTP (e.g. [8,74]).
*g*_2_: If a stimulation protocol induces a change at *other* (unstimulated) synapses onto the same postsynaptic neuron, the effect is called ‘heterosynaptic’ [66,75–77].^1^ In equation (2.2), heterosynaptic effects are described by the function *g*_2_ which does not depend on pre*_j_*, but only on the state of the postsynaptic neuron. An example of heterosynaptic plasticity is synaptic scaling [1,38] which has been modelled using a heterosynaptic term *g*_2_ with a linear weight dependence [25].
*g*_1_: If presynaptic activity alone is sufficient to induce plasticity—one could think of non-associative LTP at the parallel fibre to Purkinje-cell synapses [79,80] or spontaneous spine growth in the presence of glutamate [81]—this is captured by the function *g*_1_, which depends on the presynaptic activity pre*_j_*, but not on post*_i_*.
*g*_0_: Finally, a slow drift, a spontaneous growth or decay of the synaptic strength that does not depend on the input or the state of the postsynaptic neuron is captured by the function *g*_0_(*w_ij_*).

In our example, all terms explicitly depend on *w_ij_*. While this is not a strict requirement, it is customary to limit the allowed range of *w_ij_* to avoid infinite weight growth. As big weights are associated with physically large synapses, while the total space in the brain is limited, a bound on synaptic weights is reasonable. Depending on the implementation details, the limit can be implemented either as a ‘hard bound’ or as a ‘soft bound’ (e.g. [74,82,83]).

Virtually, all existing plasticity models can be written in a form similar to equation (2.2). Differences between model formulations arise if: (i) pre*_j_* is interpreted as presynaptic firing rate, presynaptic spikes or as chemical traces left by spikes (e.g. glutamate); (ii) post*_i_* is interpreted as postsynaptic firing rate, postsynaptic spikes, chemical traces left by postsynaptic spikes, postsynaptic calcium, postsynaptic voltage or combinations thereof; and (iii) the weight dependence is chosen identical or differently for each term. With this framework, we can now state what we mean by compensatory processes and address the question why we need them to be fast.

## 3. Why do we need rapid compensatory processes to stabilize Hebbian plasticity?

Intuitively, synaptic plasticity that is useful for memory formation must be sensitive to the present activation pattern of the pre- and postsynaptic neuron. Following Hebb's idea of learning and cell assembly formation, the synaptic changes should make the same activation pattern more likely to reappear in the future, to allow contents from memory to be retrieved. However, the reappearance of the same pattern will induce further synaptic plasticity. This forms an unstable positive feedback loop. Anybody who was sitting in the audience when the positive feedback loop between the speaker's microphone and the loudspeaker resulted in an unpleasant shriek, knows what this means. In many cases, an unstable system can be made stable by adding sensible control mechanisms [84] which are thus typically integrated in theoretically motivated plasticity models.

Let us now consider one such classic example of a learning rule. To that end, we consider Oja's rule [57]3.1
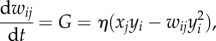
where *η* is a small constant called learning rate. As Oja's rule corresponds to a specific choice of *G* in equations (2.1) and (2.2), let us highlight the relation. First, in Oja's rule the presynaptic activity pre*_j_* is characterized by the presynaptic rate *x_j_* and the state of the postsynaptic neuron post*_i_* by its firing rate *y_i_*. Second, and with this in mind, we can now identify two terms on the right-hand side of equation (3.1). Oja's rule contains a Hebbian term *H* = *ηx_j_y_i_*, which does not have any weight dependence as well as a heterosynaptic term 
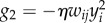
 which comes with a negative sign and is linear in the weight. Following our convention from above (equation (2.2)), we call the term heterosynaptic because it acts on all synapses, even those that do not receive presynaptic stimulation. For simplicity, and following the tradition [57], we combine Oja's rule with a linear neuron model 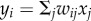.

It is quite intuitive to see how stability arises in this model. As the synaptic weights *w_ij_* grow, due to the Hebbian term, the firing rate *y_i_* of the postsynaptic neuron increases and therefore the influence of the negative heterosynaptic term gets stronger. Because the heterosynaptic term has a superlinear dependence on *y_i_*, it is guaranteed to ‘catch up’ with the Hebbian term eventually. It can be shown that for a linear neuron model and sufficiently small *η*, Oja's rule ensures that the weights converge such that 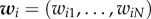 aligns with the first principal component of the data *x*, while the squared sum of all afferent weights remains normalized [57].

We interpret the heterosynaptic term in Oja's rule as RCP. First, it is rapid because it responds instantaneously to activity fluctuations in *y_i_*. Second, it is compensatory because it ensures stability by effectively enforcing a constraint on the afferent synaptic weights [10,57]. Biologically, such a heterosynaptic effect could be obtained, for instance, when synapses have to compete for a shared resource [57,85] or send chemical signals to each other [86].

One could now ask if we really need these compensatory processes to be rapid. Could we not simply replace the instantaneous firing rate *y_i_* in the heterosynaptic term by a slower variable? The timescale of the slow variable could be related in a biological system to the time necessary to estimate the firing rate from, e.g. calcium concentration, and translate these into metaplastic changes in the learning rule. To illustrate the general idea by a concrete example, we take Oja's rule, as in equation (3.1), except that, in the heterosynaptic term, we replace 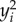 by 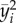, where 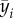 is a low-pass filtered version of the postsynaptic rate3.2
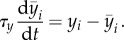


**Figure 2.**
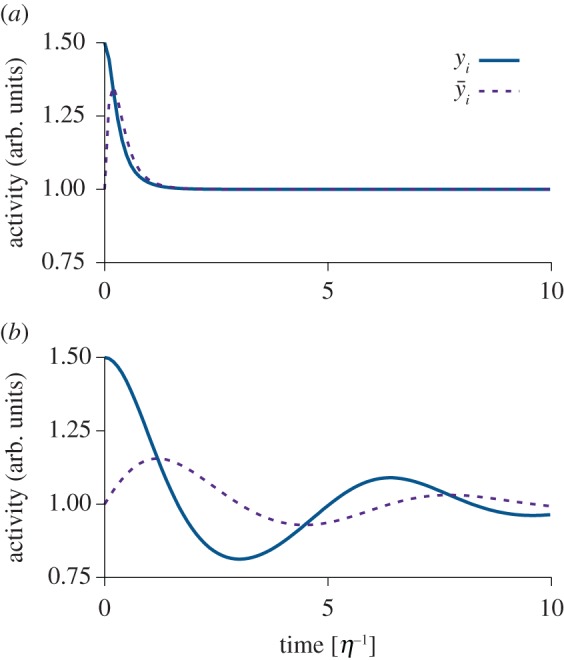
Illustration of rapid and slow compensatory processes in a variation of Oja's rule. (*a*) Here, we have used a fast filter time constant *τ* = 0.1 *η*^−1^ (cf. equation (3.2)) and plot the output firing rate *y_i_* (solid) and the delayed estimate 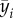 (dashed). (*b*) Same as in (*a*), but with *τ* = 2*η*^−1^. Model: we simulated 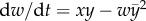, 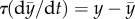 and *y* = *wx* with *x* ≡ 1.

If we choose *τ_y_* = 1 ms (for a fixed *η* of, e.g. *η^−^*^1^ = 10 ms), the processes 
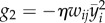
 would still be considered as rapid (figure 2*a*), but if we choose *τ_y_* = 1 h, it would be considered as slow. When the compensatory processes are too slow, positive feedback induced by the Hebbian term is prone to take over and oscillations (figure 2*b*) or even runaway dynamics arise. This is why we generally want the compensatory processes to be *rapid*.

**Figure 3.**
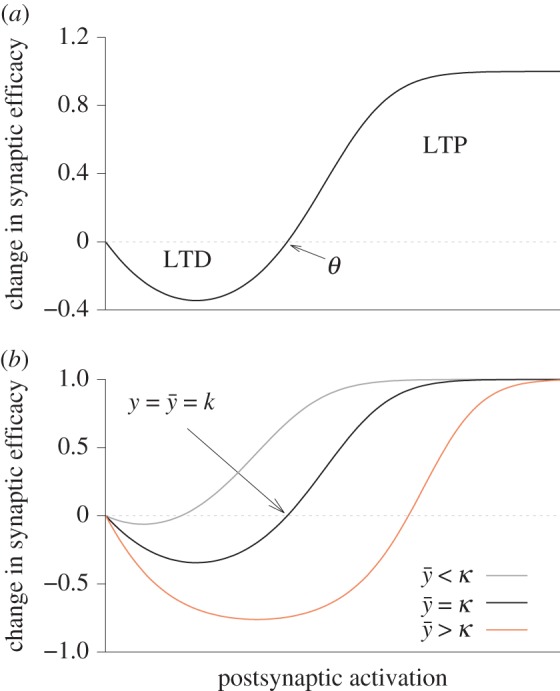
Most plasticity models can reproduce the notion of a plasticity threshold reported in experiments. (*a*) The change in synaptic efficacy in many plasticity models is a function of variables related to postsynaptic activation. The parameter *θ* is fixed and marks the transition point (threshold) between LTP and LTD. (*b*) Schematic of the action of the homeostatic moving threshold *θ*(*t*) in the BCM model [5]. When the average 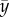 is larger than the target value *κ*, *θ*(*t*) shifts to higher values. Likewise, *θ*(*t*) shifts to lower values when 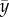 is too low. For 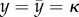 changes in synaptic efficacy are zero.

The same problematic has also been demonstrated in the Bienenstock–Cooper–Munro (BCM) model [5]3.3
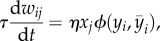
where *ϕ* is a nonlinear function with a shape characterized by a threshold *θ* between LTP and LTD (figure 3*a*) consistent with some induction protocols [70,87]. The threshold *θ* depends on the moving average 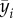 over past neuronal activity (figure 3*b*) where 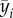 is defined in equation (3.2). This is the reason why the model is said to have a ‘sliding’ threshold.

To ensure stability, the BCM model requires two independent assumptions. First, the sliding threshold has to be a superlinear function of 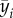 [5]. A standard choice is [88]3.4
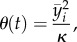
where *κ* is the ‘target rate’ to which the moving average of the postsynaptic firing rate should converge. Second, *τ_y_* cannot be ‘too large’ compared with *τ*, because otherwise oscillations or runaway activity occur [17,26,88]. In fact, the ratio *τ_y_*/*τ* determines the stability of the model.

Oscillations and instabilities are generic to many nonlinear systems and not limited to the above models. Control theory enables theoreticians to identify parameter ranges that lead to stable behaviour and avoid instabilities [25,84,89]. The control theoretic analysis of several plasticity models relying on moving averages of the postsynaptic firing rate shows that the response timescale of the compensatory processes is constrained from above [17,26,88,90]. In other words, the response time of the firing rate control has to be ‘relatively fast’ compared with Hebbian plasticity. But how fast is fast enough? Is it seconds, hours or days?

## 4. How fast do compensatory processes have to be?

Because time can be rescaled arbitrarily in the above model, a quantitative answer to the question can only be given for a specific combination of neuronal, network and plasticity model parameters once units of time are calibrated with biological data. In other words, we need to put a numerical value on *τ* to set the timescale of *τ_y_*. To fix a timescale,^2^ one can thus use any quantitative plasticity model which has been fitted to experimental data in combination with plausible spiking neuron models embedded into a spiking neural network with a biologically inspired activity state.

Such an analysis was done by Zenke *et al*. [26] using the plasticity model of Pfister & Gerstner [27], combined with negative feedback via either a sliding threshold or synaptic scaling. The critical timescale *τ*_crit_ was determined as the value *τ_y_* (equation (3.2)) above which a recurrent neural network, initially tuned to a low-activity asynchronous state [91,92], generates runaway activity. Using simulations and mean field theory, *τ*_crit_ was found to be of the order of seconds to minutes. Thus, the negative feedback needs to be too rapid to be linked to the known experiments of homeostatic synaptic plasticity reviewed in figure 1.

Several remarks are in order. First, although signatures of the stereotypical activity dependence of the BCM model (figure 3*a*) are also present in STDP data and captured by many modern plasticity models [27,61–63,65,93], the existence of a sliding threshold mechanisms is still a topic of ongoing debate. However, we have shown analytically, and confirmed in simulations, that the instability that arises through slow feedback in the BCM model is virtually identical to the situation in which the sliding threshold in equation (3.3) is replaced by a fixed threshold and instead synaptic scaling is added to the model [26]. Additionally, the analysis suggests that similar temporal requirements hold for an entire family of plasticity models with an explicit rate dependence (see Yger & Gilson [90] for a review). Note, however, that additional instabilities can arise in the case of synaptic scaling [89].

Second, the critical timescale *τ*_crit_ depends not only on the plasticity model, but also on multiple parameters of the neuron and network model. Moreover, the results showed a strong dependence on background firing rate which was comparatively high in the Zenke *et al*. [26] model (approx. 3 Hz). On the other hand, robust stability is only possible if the actual value of *τ_y_* is chosen to be much smaller than *τ*_crit_. The precise value of the critical timescale has therefore to be viewed with care: we believe that any published numerical value for *τ*_crit_ may be out by a factor of 5 or 10 (because of uncertainty in choices of neuronal and network parameters), but it is unlikely to be out by a factor of 100. In any case, despite the remaining uncertainty, these numerical results suggest that most experimental forms of homeostatic plasticity are too slow to stabilize Hebbian plasticity as captured by current models, and that RCPs are required to maintain stability.

A recent voltage-based plasticity model by Jedlicka *et al*. [23] corroborates the above findings. By fitting their model with a rapid sliding threshold to *in vivo* data from dentate gyrus, the authors find *τ_y_* ≈ 12 s which allows them to speculate that the sliding threshold could be linked to CaMKII inactivation.

Interestingly, Toyoizumi *et al*. [17] arrive at qualitatively similar conclusions by carefully analysing the different phases of synaptic dynamics following monocular deprivation [35]. Specifically, they find that a fast sliding threshold guarantees stability, but provides a poor fit to experimental data, whereas as slow sliding threshold compromises stability altogether. Consequently, they suggest a model in which LTP and LTD saturate quickly to attain steady states. Additionally, a slow form of homeostatic plasticity is required to capture the data (cf. figure 1), but is no longer required to provide stability. In their model, LTP and LTD saturate due to soft weight bounds. However, the model does not crucially depend on this point and would presumably also work with other RCPs.

Finally, these findings are in good empirical agreement with many existing simulation studies of plastic network models (figure 1)—in each of these, a rapid homeostatic control mechanism on a timescale of seconds to minutes was implemented to maintain stability [14,19,21,22,27,63,94].

We can summarize our insights as follows. The fact that Hebbian plasticity has to be appropriately constrained through stabilizing mechanisms to avoid runaway activity is well known. Classic models such as Oja's rule or the BCM model, for example, explicitly include appropriate mechanisms based on the postsynaptic firing rate as an indicator and driver of stabilizing processes. However, the fact that these processes have to be rapid in absolute terms only becomes apparent when units of time are fixed to a biologically meaningful timescale. Moreover, RCPs need to be even more rapid in large recurrent network models, because a large number of plastic synapses tend to amplify instabilities unless the learning rate is scaled with the inverse number of synapses. Accumulating evidence suggests that biological forms of LTP and LTD have to be accompanied by RCPs which operate on timescales of seconds to minutes and are thus orders of magnitude faster than most known forms of homeostatic plasticity (cf. figure 1). This answers the questions as to why RCPs are needed and gives us first upper limits on the intrinsic timescale of RCPs to successfully stabilize LTP and LTD. However, do we want RCPs to be a rapid version of homeostatic plasticity with a single set point? We will now discuss this question in more detail, before we turn to potential mechanisms.

## 5. Functional consequences of enforcing constraints on short timescales

Homeostatic mechanisms are typically interpreted as negative feedback processes [41], which rely on an error signal to maintain a given control variable of a dynamical system at designated target values, or set points. Many control systems have a single set point. For instance, an autopilot tries to maintain a single given course at any given time. Similarly, most models of homeostatic plasticity have a single target value, such as the average postsynaptic firing rate (see *κ* in the BCM model equations (3.3) and (3.4)). Suppose we are dealing with a fast negative feedback processes, what are the functional consequences for plasticity and circuit dynamics? To do so, we focus on commonly found forms of firing rate homeostasis (FRH) with a *single* firing rate set point [15,16,95].

Neurons encode information in changes of their electrical activity levels. For instance, subsets of simple cells in the visual system fire spikes in response to specific edge-like features in the visual field [96]; cells in higher brain areas respond with high specificity to complex concepts and remain quiescent when the concept they are coding for is not brought to mind [97–99]; and finally certain neurons respond selectively with elevated firing rates over extended periods during working memory tasks [100–102]. The ability of neurons to selectively indicate through periods of strong activity the presence of specific features in the input or specific concepts in working memory is an important condition for computation.

**Figure 4.**
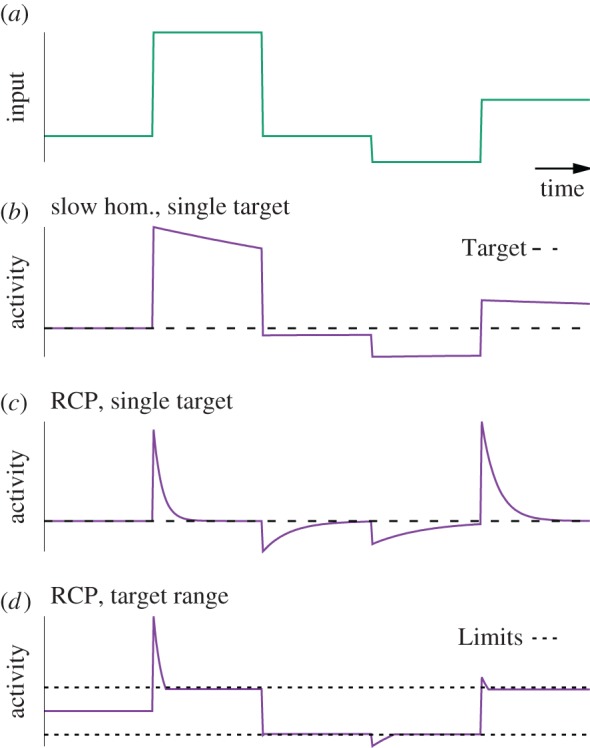
The effect of slow homeostatic mechanisms and RCPs on the neuronal code. (*a*) Fluctuating external world stimulus over time. (*b*) Neural activity ‘*A* = *w* × input’ over time. A slow negative feedback mechanism (homeostasis) adjusts *w* to push *A* towards a single target (dashed line) which is rarely reached; negative feedback is modelled as 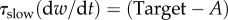. With a slow homeostatic mechanism, a neuron can track input relatively accurately. (*c*) Same as *b*, but for a rapid compensatory process (*τ*_slow_ = 50*τ*_fast_) which drives the activity quickly to the target value. If the timescale of feedback is comparable to that of the stimulus, negative feedback interferes with the neuron's ability to track the stimulus. (*d*) RCPs enforcing an allowed range (limits indicated by dotted lines). Even though the neuronal activity does not capture all the diversity of the input, it does capture some of it. Here, we modelled the RCPs as the following nonlinear extension of the above model: 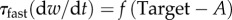 with *f*(*x*) = *x* for 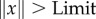 and *f*(*x*) = 0 otherwise.

**Figure 5.**
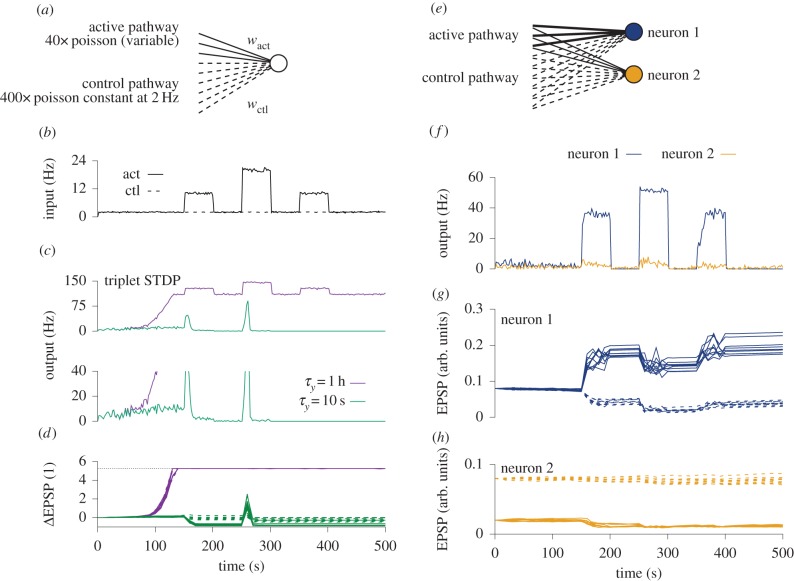
Firing rate stabilization, synaptic weights and feature selectivity through RCPs. (*a*) Schematic figure of single neuron with two distinct input pathways. The ‘active’ pathway consists of 40 Poisson neurons switching their rates synchronously among 2, 10 and 20 Hz. The control pathway consists of 400 neurons all firing constantly at 2 Hz. Synaptic plasticity is modelled with triplet STDP [27] with a BCM-like sliding threshold as defined in equations (3.2)–(3.4). All weights are initialized at the same value and can freely move between hard bounds at zero and ≈6 times the initial value. (*b*) Population firing rates of the input populations averaged over 2 s bins. Firing rates in the active pathway (solid line) are switched three times from 2 Hz to a higher rate and back (10, 20 and 10 Hz for 50 s each time), whereas firing rates in the control pathway are constant at 2 Hz. (*c*) Output firing rates of a single postsynaptic neuron. Purple, slow sliding threshold, with time constant *τ_y_* = 1 h; green, fast sliding threshold, *τ_y_* = 10 s; (see Zenke *et al*. [26], *κ* = 3 Hz in equation (3.4)). Top and bottom show the same firing rate plot for different *y-*axis ranges. (*d*) Relative weight changes for 10 randomly chosen weights from each pathway for the slow (purple) and the fast (green) sliding threshold. Solid lines correspond to active pathway weights and dashed lines to the control pathway. Note that for the fast sliding threshold the active pathway develops weaker synapses than the control pathway. For the slow sliding threshold, all weights saturate. (*e*) Simplified sketch of the same set-up as in (*a*), but with two postsynaptic neurons. The only difference between the two neurons is the choice of initial conditions of the synaptic weights. For neuron 2, the active pathway weights are initialized at a lower value than for neuron 1. All synaptic weights exhibit triplet STDP combined with heterosynaptic plasticity [103]. (*f*) Output firing rates of the two neurons over time. Neuron 1 (blue) responds selectively (with rates more than 30 Hz) to the elevated inputs in the active pathway (cf. *b*). Neuron 2 (orange) continues to fire with low firing rates. (*g*) Evolution of weights over time for neuron 1. Active pathway weights are plotted as solid lines and control pathway weights are dashed. For neuron 1, the synapses in the active pathway undergo LTP during the first strong stimulation of the active pathway. However, weights quickly saturate. Synapses in the control pathway exhibit heterosynaptic depression. (*h*) Same as *g*, but for neuron 2. The weights in the active pathway are slightly depressed during initial stimulation.

Is the notion of a single set point compatible with the task of neurons to selectively respond to stimulation? If negative feedback control of firing rates is slow (e.g. synaptic homeostasis), neuronal firing can deviate substantially from the mean firing rates during short times and thus encode information (figure 4*a,b*). However, we have strong reasons to believe that a slow homeostatic control mechanism cannot stabilize the ravaging effects of Hebbian plasticity. So what can we say about a putative RCP? If it were to act like FRH, but on a short timescale (e.g. seconds to minutes), neural codes based on neuronal activity become problematic because synaptic plasticity starts to suppress activity fluctuations which could be carrying important information (figure 4*c*). For example, if the RCP has a timescale of 2 s, rapid stimuli that change on a timescale of 0.5 s would be transmitted as a rate signal of the postsynaptic neuron, while stimuli sustained for more than 5 s would be suppressed by compensatory synaptic changes. Even more alarmingly, certain forms of homosynaptic plasticity, like the BCM [5] or the triplet STDP [27] model endowed with a rapid sliding threshold, not only suppress high-activity periods, but also ‘unlearn’ previously acquired selectivity and erase memories (figures 4*a,c* and 5*a–d*). Therefore, RCPs which enforce a single set point are hardly desirable from a functional point of view. Thus, the requirement of fast negative feedback control over Hebbian plasticity with a single set point poses a problem in itself.

It is important to appreciate that this problem arises from the combination of a *single* target with the requirement to implement negative feedback on a short timescale. Fortunately, most forms of homeostatic plasticity are slow (cf. figure 1). Thus, known homeostatic mechanisms do not interfere with neuronal coding. For RCPs not to interfere with coding either, it thus seems important that they do not enforce a single set point constraint on postsynaptic activity. Nevertheless, these RCPs have to prevent runaway activity.

There could be at least one simple solution to this conundrum [17,103]. Suppose there are two, or more, set points enforced by two or more RCPs. For instance, one RCP could activate above a certain activity threshold and ensure that neuronal activity does not exceed this threshold. Similarly, a second mechanism could activate below another lower activity threshold. The combined action of the two mechanisms enforces neural activity to stay within an allowed range, but still permits substantial firing rate fluctuations inside that range (figure 4*d*).

When such a pair of RCPs is combined with a form of Hebbian plasticity which has its plasticity threshold within the limits of the allowed activity regime, the neural activity of the compound system naturally becomes multistable for prolonged stimulation with the same stimulus. Within the allowed range no RCP is active, but Hebbian plasticity is intrinsically unstable. Thus, for a stimulus sustained longer than the timescale of the RCP and Hebbian learning, any value of the postsynaptic rate within the allowed region will lead to either LTP or LTD until the system reaches the limits at which either RCP rapidly intervenes by undoing any excess LTP or LTD from there on. The compound system exhibits therefore two, potentially stimulus dependent, stable equilibrium points, one at low and one at high activity.

Let us apply these considerations to two different systems, a sensory system and a memory system. To be concrete, we assume that in either system the timescales of both LTP induction and RCP are 2 s. In the sensory system, each neuron will respond in a graded manner to short stimuli (say, with a duration of half a second) because synapses hardly change during a single stimulus duration. However, the repeated stimulation with different stimuli will cause long-lasting weight changes. The location of the high-activity fixed point depends on the stimulus ensemble used during stimulation. Moreover, if we drive the neuron with a single sustained stimulus, the high-activity fixed point adjusts on the timescale of a few seconds and reflects the value of the input.

The case of a memory system was considered in [103]. Suppose the high-activity fixed point corresponds to a memory retrieval state, while the low-activity equilibrium is associated with a quiescent memory which is not being recalled. Because both states are stable, it is irrelevant whether the memory is recalled every other minute or once a year. Importantly, this is different from models with rapid FRH, which might require neuronal activity to regularly turn on and off to satisfy the constraint. An example for this is the network model by Litwin-Kumar & Doiron [14] in which inhibitory synaptic plasticity (ISP) acts as rapid FRH with a single activity fixed point.

We can therefore answer the third of the questions raised in §1: ‘The functional consequences of a rapid control mechanism with a single set point are that neurons lose the flexibility that is necessary for coding’. The consequences are therefore undesirable and the proposed solution is to design RCPs that allow for several set points or a target range of permissible activity. In the next section, we will first discuss common ways to constrain unbounded weight growth and explain why they are insufficient to provide stability, before we turn to potential candidate plasticity mechanisms which could act as RCP. Finally, we show an example of a spiking network model based on these principles which forms and recalls memories encoded in cell assemblies.

## 6. Potential mechanisms

To stabilize Hebbian plasticity, any RCP at the synaptic, dendritic, neuronal or network level can be considered. Owing to temporal and spatial constraints of the biological substrate it seems most probable, however, that the fastest mechanisms are implemented as close to the synapse as possible.

At the synaptic level, excessive growth has traditionally been limited by soft or hard bounds on individual synaptic weights or other choices of explicit weight dependence of the Hebbian and heterosynaptic terms in equation (2.2) [8,10,25,65,82,83,104–107]. For example, to avoid bimodal weight distributions, which sometimes arise in competitive models, but are not observed in biology, a range of multiplicative plasticity models [25,82,83,104,105,108], with an appropriate choice of the weight dependence of *H* in equation (2.2), have been devised. However, bounds on individual synaptic weights only impose an implicit constraint on the postsynaptic activity. To see this, consider a permissible range of individual synaptic strength of, say, ±50% around the initial efficacy, which seems not uncommon for plasticity induction protocols. However, by setting this range we do not automatically exclude the situation in which *all* synapses increase their efficacy by 50% which would in all likelihood correspond to pathological activity levels.

To avoid such runaway activity, plasticity has to ensure that not all synapses are potentiated or depressed equally. Rather there should be some form of competition which ensures that when one set of synapses is potentiated other synapses are depressed by a certain amount. While some degree of competition can be seen in STDP models, in which presynaptic spikes compete in time to elicit a postsynaptic spike [8,58,109,110], this competition is generally weak [25] and without additional constraints, activity levels still succumb to runaway effects with detrimental consequences in recurrent neural networks [14,26,103]. Robust competition, for instance through a BCM-like threshold [5], or explicit constraints on the sum of weights [10], is therefore of paramount importance for plasticity models.

In summary, there exist multiple mechanisms to limit growth of individual synaptic weights. However, to achieve robust synaptic competition and stability of output firing rates, more explicit activity constraints are required, as exemplified in the BCM model, or through explicit heterosynaptic interactions, similar to Oja's rule (cf. equation (3.1)). We have already argued that these constraints need to be enforced rapidly. We now ask what possible mechanisms at the neuronal or network level could achieve that.

At the network level, RCPs might be implemented by inhibition and ISP which could influence plasticity at excitatory synapses either directly or indirectly. Some theoretical forms of ISP are known to implement a rapid form of FRH for individual neurons [14,111]. With accumulating experimental evidence for ISP [95,112,113], it therefore seems likely that synaptic inhibition influences plasticity of excitatory synapses at least indirectly through changes in activity. However, in experiments, the timescale of FRH mediated through ISP appears to be relatively slow [95] and it remains to be seen whether biological forms of ISP can act as RCPs or whether they have a rather homeostatic role.

However, in some cases, inhibition without ISP can have a stabilizing effect. Lim *et al*. [114] have recently demonstrated that this can indeed lead to stability of certain forms of Hebbian plasticity. Moreover, inhibition can also directly affect and regulate excitatory plasticity [115]. Particularly interesting in this context are results by Delgado *et al*. [116], who observed total-conductance-dependent changes of the STDP curve depending on excitatory and inhibitory background input. Their results suggest that increased, but balanced, excitatory and inhibitory input biases the STDP window towards LTD and can thus act as a RCP. Delgado *et al*. [116] demonstrated this in a single-neuron feed-forward model, but it is not yet known whether these results generalize to larger networks.

At the neuronal level, algorithmic normalization of afferent synaptic weights is a commonly used mechanism to stabilize Hebbian plasticity in network models while simultaneously allowing structure formation [9,10,13,14]. While such rapid and precise scaling at the neuronal level has been criticized as biologically implausible [5], an ‘approximate’ scaling could potentially be achieved through heterosynaptic plasticity at the dendritic level [117].

Heterosynaptic plasticity has moved back in focus recently because of its potential role as an RCP [39,40,86,103,118]. Importantly, some forms of heterosynaptic plasticity are fast, and provide primarily negative feedback in response to high postsynaptic activity levels [39,119] or in the presence of strong LTP on a dendritic segment [86]. This is reminiscent of Oja's rule (equation (3.1)) and seems well suited to counteract runaway LTP. In contrast to Oja's rule, these heterosynaptic changes are induced by bursts of postsynaptic activity which implies that the quadratic term 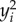 in equation (3.1) should be replaced by a term that is triggered either by firing rates *y_i_* above some threshold [118] or by a higher power such as 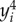 [103].

In models which also show runaway LTD at low activities (e.g. [27,63]), an additional RCP is needed which either saturates or counteracts LTD. Possible forms of plasticity include, but are not limited to, transmitter-induced plasticity, homeostatic scaling-up or spontaneous spine formation.

In the following section, we review a plasticity model which combines Hebbian plasticity with two RCPs that enable more than a single set point of neuronal activity. We also discuss, in the context of the model, the potential role of additional slow homeostatic mechanisms.

## 7. Learning and recall in a recurrent spiking network model

We now discuss a learning rule which combines a plausible model of Hebbian plasticity with two additional RCPs [103]. For sensible combinations, this compound model does not suffer from the runaway effects of purely Hebbian plasticity and exhibits intrinsic multistability instead (cf. figure 4*d*).

The basic logic of multistable plasticity can be summarized as follows. At high activity levels, a rapid form of heterosynaptic plasticity limits runaway LTP and creates synaptic competition. Similarly, at low activity levels, an unspecific form of plasticity which only depends on presynaptic activity prevents runaway LTD. The well-orchestrated interplay between these adversarial plasticity mechanisms dynamically creates multistability of neuronal activity and prevents pathological runaway effects.

Our approach is quite general and many Hebbian plasticity models can be stabilized through the addition of two non-Hebbian forms of plasticity. For illustration purposes, we now focus on the triplet STDP model for which biologically plausible sets of model parameters exist [27]. To prevent runaway LTP, we require a mechanism which balances out potentiation at high activity levels. To that end, we use a form of weight-dependent, multiplicative heterosynaptic depression [40,103,118]. Our choice of a purely heterosynaptic RCP is one possibility, but other homosynaptic forms of plasticity could achieve similar results. For instance, ‘heterosynaptic’ LTD [23,120] which requires low presynaptic activity for depression in the unstimulated pathway, is one possibility. In short, as long as LTP in a strongly stimulated pathway is accompanied by either ‘no change’ or synaptic depression of synapses with low levels of presynaptic activity, runaway LTP can be avoided. To similarly prevent runaway LTD in our model, we introduced a hypothetical form of transmitter-induced plasticity. Together the three plasticity mechanisms—Hebbian plasticity and two RCPs—work in symphony to generate stable levels of neuronal activity.

Let us consider the weight *w_ij_* from a presynaptic neuron *j* to a postsynaptic neuron *i*. Although the full model is an STDP model, we now express its core ideas in terms of a rate model of the pre- and postsynaptic rates *x_j_* and *y_i_*:7.1
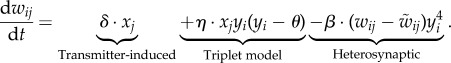


Here, *δ* and *β* are strength parameters for the two non-Hebbian components of the plasticity model; *η* is the strength parameter (learning rate) for Hebbian plasticity; and 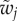 serves as a reference weight that can be related to consolidation dynamics [54,103]. Note, because the negative feedback plasticity mechanisms are ‘rapid’, there is no low-pass filtered variable 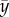 in this expression. Runaway effects which normally would occur in the triplet STDP model without RCPs (figure 5*a–d*) are avoided with the additional plasticity mechanisms. Owing to its rapid action and the high power, the heterosynaptic term in equation (7.1) acts as a burst detector [118] which dominates at high activity levels and prevents LTP runaway dynamics (figure 5*e,f*).

For sensible choices of *δ* and *β*, neuronal firing rates remain in intermediate regimes (figure 5*f*) and synaptic weights in the model converge towards stable weights 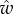 whose values are dependent on the activation history and enable the formation of long-term memories (figure 5*g*). Importantly, the model preserves the plasticity threshold between LTD and LTP of the original triplet STDP model. The triplet STDP model together with the non-Hebbian plasticity mechanisms, dynamically creates one unstable and two stable equilibrium points. The activity level of the higher stable fixed point depends on the stimulus. In particular, it is sensitive to the number, firing rate and temporal structure of the active synaptic inputs and a stronger stimulus will typically result in a higher steady-state response. For any given stimulus, synaptic weights converge rapidly towards one of two possible stable equilibrium states (figure 5*f–h*). First, there is a ‘selective’ equilibrium state associated with high postsynaptic activity. In this state, some weights are strong while other weights onto the same postsynaptic neuron remain weak. Thus, the neuron becomes selective to features in its input (neuron 1 in figure 5*f*,*g*). Second, there is a ‘non-selective’ equilibrium at low activity (figure 5*f*,*h*). Which fixed point a neuron converges to depends on its initial conditions, lateral interactions and the details of the activation pattern (figure 5*g,h*). Once weights have converged to one of the respective stable states, they keep fluctuating, but do not change on average. As the RCPs do not impose a single set point, activity patterns are not unlearned when a certain input is kept active (inactive) for extended periods of time (compare figure 5*d* with 5*g,h*).

As a result, neuron 2 shown in figure 5*h* shows ‘no learning’ which seems undesirable at first. However, it is in fact useful for such a stable equilibrium to exist when learning is considered as a network phenomenon. Other neurons (neuron 1 in our case) already are selective and code for a given stimulus. Analogously, neuron 2 might in fact code for a different stimulus which is not active at the moment, in which case we would like to perturb it as little as possible while other neurons ‘learn’ (figure 5*h*). Similar dynamics can be achieved in learning models with strong lateral inhibition which completely suppresses neuronal activity and thus also associative plasticity. In the present scenario, however, this is not the case. Neuron 2 is still firing with some low background activity throughout (figure 5*f*).

**Figure 6.**
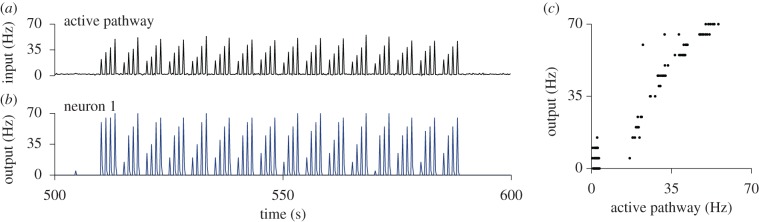
Reproducible responses to input stimuli with varying strength. (*a*) Population firing rate of active input pathway for the continuation of the simulation shown in figure 5*b,e–h*. The neuron is stimulated 16 times with four interleaved steps of increasing input firing rate in the active pathway. (*b*) Output firing rate of neuron 1 (200 ms bins; cf. figure 5*f*). After the first set of stimuli, responses to the later sets remain graded and are overall reproducible. (*c*) Data points from (*a*) and (*b*) (input and output) plotted against each other.

There are several aspects worth noting about the model. First, heterosynaptic plasticity not only stabilizes Hebbian plasticity in the active pathway, it also introduces synaptic competition between the active and the control pathway (figure 5*g*). In contrast to BCM-like models in which heterosynaptic depression of the inactive pathway depends on intermediate periods of background activity in between stimuli [23,88], here the heterosynaptic depression happens simultaneously with LTP induction (figure 5*g*). Second, although the learning rule effectively implements a rapid redistribution of synaptic weights reminiscent of synaptic scaling, it is still a fully local learning rule which only depends on information that is available at the synapse (cf. equation (7.1)). Third, although the learning rule effectively implements bistable dynamics for each stimulus, the ‘selective’ equilibrium level remains stimulus dependent, which allows the neuron to respond in a graded and reproducible way to input stimuli of varying intensity (figure 6). Fourth, in general the reference weight 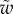 is not fixed, but follows its own temporal dynamics on a slower timescale (≈20 min and more). Such slow complex synaptic dynamics are essential to capture experiments on synaptic consolidation [50,54,121], but could similarly be used to model slow forms of homeostatic plasticity [17].

**Figure 7.**
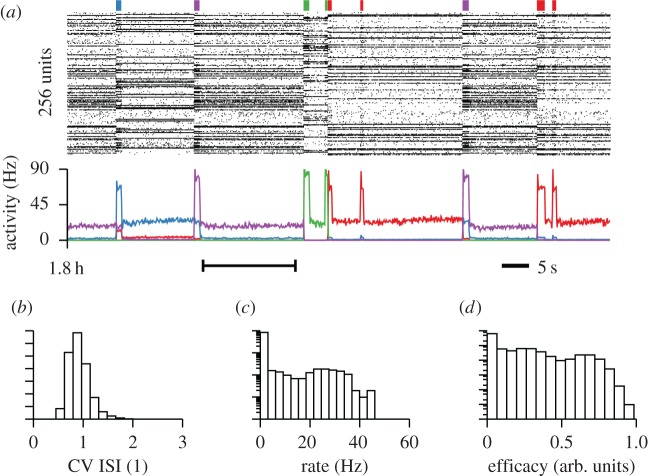
Stable activity in a recurrent neural model with ongoing plasticity. (*a*) Memory recall in associative cell assemblies through selective delay activity in a network which previously has learned to distinguish between four repeating input patterns [103]. The coloured bars at the top indicate time and duration of external stimulation with one out of four stimuli. The colour indicates stimulus identity. The spike raster in the middle shows spiking activity of 256 randomly chosen excitatory cells from the network. The graph at the bottom shows the firing rate of the four subpopulations defined by the cell assemblies in the network. The multistable plasticity model of equation (7.1) is active throughout the simulation. (*b*) Histogram of the coefficient of variation of the inter-spike interval of excitatory cells in the network during the interval indicated by the black range bar in (*a*). (*c*) As in (*b*), but for mean neuronal firing rates over the same interval. (*d*) Distribution of synaptic efficacies of plastic recurrent synapses at the end of the network simulation. Figure adapted from Zenke [122] and Zenke *et al*. [103].

Finally, the stability properties of the learning rule in equation (7.1) are not limited to simple feed-forward circuits, but generalize to more realistic scenarios. Specifically, the combination of heterosynaptic and Hebbian plasticity enables stable online learning and recall of cell assemblies in large spiking neural networks (figure 7*a,b*; [103]). Stationary firing rates in the model depend on the connectivity pattern and the spiking statistics of active inputs. In a recurrent network, however, output spike trains pose as inputs to other neurons. As a non-trivial consequence, stationary solutions of the network state exhibit firing rate distributions which are unimodal and long-tailed (figure 7*c,d*). Individual neuronal firing rates only stabilize under stationary conditions. If the rates are non-stationary, for instance, owing to the inclusion of additional adaptation processes in the neuron model, rates in the model drift on behavioural timescales (see Zenke *et al*. [103] for details).

In summary, orchestrating Hebbian plasticity and RCPs on comparable timescales dynamically generates multistability. This reconciles the experimentally observed fast induction of synaptic plasticity with stable synaptic dynamics and stability of learning and memory at the single neuron level as well as in large networks. However, there are a few caveats with this approach which we will discuss in the following section.

## 8. Problems of rapid compensatory processes at the single neuron level

Consider a population of neurons with plastic synapses which follow intrinsically stable plasticity dynamics such as the ones described in §7. To encode and process information efficiently, neuronal populations need to create internal representations of the external world. Doing this efficiently requires the response to be sparse across the population. In other words, only a subset of neurons should respond for each stimulus. Moreover, different stimuli should evoke responses from different subsets of neurons within the population to avoid all stimuli looking ‘the same’ to the neural circuit. Finally, individual neurons should respond sparsely over time. Imagine a neuron which is active for all possible stimuli. It would be as uninformative as one which never responds to any of the inputs. Therefore, to represent and process information in neural populations efficiently, different neurons in the population have to develop selectivity to different features.

Multistable plasticity at the neuronal level as described above does not prevent neurons from responding weakly to all stimuli (see, for example, Neuron 2 in figure 5*f*). This is a direct consequence of the fact that the model presented here does not have a sliding threshold like the BCM model. Moreover, with more similar initial conditions and in the absence of lateral inhibition both Neurons 1 and 2 could have developed selectivity to the *same* input. Thus, in a large network in which all synapses are changed by the intrinsically stable plasticity rule introduced above, all neurons could end up responding to the same feature. How can such an undesired outcome be avoided?

To successfully implement network functions like the ones shown in our example (figure 7), several network parameters and properties of the learning rules themselves need to be fine-tuned and maintained in sensible parameter regimes. For instance, successful learning as demonstrated by Zenke *et al*. [103], depends on sensible choices of the initial synaptic weights and connectivity values. To achieve the necessary degree of tuning and maintenance, biological networks presumably rely on additional forms of plasticity which drive the network towards a dynamical state which is conducive for learning. However, due to the intrinsic stability of the learning rule, these additional mechanisms, for instance, a BCM-like sliding threshold, can now safely operate on much longer timescales. This suggests that homeostatic plasticity and metaplasticity could fulfil this fine-tuning and maintenance role.

## 9. What is the role and scope of slower homeostatic plasticity mechanisms?

Diverse homeostatic mechanisms exist in the brain at different temporal and spatial scales [123–127]. We have argued that RCPs are important for stability, but what advantages do slow homeostatic mechanisms have and what is their computational role?

An advantage of slow homeostatic processes is that they can integrate activity over long timescales to achieve precise regulation of neural target set points [25,38,89]. Longer integration times also allow integration of signals from other parts of a neural network which take time to be transmitted as diffusive factors [124,128]. Slower homeostasis thus seems well suited for control problems which either require fine-tuning or a spatially distributed homeostatic regulation of functions at the network level.

There are at least two important dynamical network properties which are not directly controllable by neuronal-level RCPs (equation (7.1)). First, temporal sparseness at the neuronal level is not automatically guaranteed. A neuron that never responds to any stimulus will never learn to do so under multistable plasticity if the LTP threshold is too high. Similarly, a neuron that always responds is uninformative, but will not change its behaviour if the LTD threshold is too low. Second, spatial sparseness at the network level, in the sense that for any stimulus a subset of neurons responds, is also not automatically guaranteed. Lateral inhibition is a suitable candidate to decorrelate responses of different neurons in a network, but, as excitatory synapses change during learning, the strength of lateral inhibition needs to be co-regulated.

The problem of *temporal sparseness* can be solved by any mechanism which ensures that a neuron which has been completely silent for very long, eventually ‘gets a chance’ to reach an activity level above the LTP threshold. This can be achieved by either lowering the threshold as in the BCM theory [5,88,103] or by slowly increasing the gain of either the neuron itself or the excitatory synapses through other forms of slow homeostatic plasticity [17,129,130]. Finally, similar homeostatic effects could be achieved by dis-inhibition through the action of neuron specific ISP [112] or by decreasing the response of inhibitory neurons [33,131]. Conversely, a neuron that is uninformative because it is always active could decrease its response to some stimuli by the opposite action of one or several of the homeostatic mechanisms mentioned above, such as increased inhibition, reduced excitation, or reduced excitability.

While it is conceivable that mechanisms addressing the issue of temporal sparseness could act locally at the neuronal level, it is clear that enforcing *spatially sparse activity* at the population level can only be achieved in a non-local manner. A common approach to guarantee spatial sparseness in models is to include lateral inhibition, as done in subspace learning algorithms [132,133], sparse coding paradigms [134,135], or models of associative memory [14,103,136–140]. However, achieving appropriate levels of inhibition can be difficult, especially if excitatory synaptic weights are not static, but change over time and on a per neuron basis [111]. To solve this task in biological networks, ISP would be a natural candidate. However, most existing models of ISP are purely local and tune inhibition on a per neuron level [111,112,141]. More specifically, ISP acts as a neuronal RCP which rapidly drives firing rates to a single stable set point (cf. figure 4) [111]. To achieve a certain level of spatial sparseness through any form of homeostatic plasticity, requires a signal with a wider scope which encodes network activity [124,128]. Using such a signal, it is then possible to modulate plasticity [73]. For example, in Zenke *et al*. [103] ISP is modulated by a low-pass filtered signal which encodes network activity. As a result, the combination of intrinsically multistable plasticity at excitatory synapses and ISP, ensures that recurrent inhibition is tuned to a level where only one cell assembly can be active at any given time. Importantly, this homeostatic mechanism does not have to be permanently active. For instance, once the inhibitory feedback within the model is tuned to the ‘sweet spot’ at which the network can operate, ISP can be turned off safely without impairing stability. Similarly, it seems likely that some forms of homeostatic plasticity could be dormant for most of the time and spring into action only during the initial phase of development [142] or when an extreme external manipulation changes the network dynamics [1].

We are thus able to answer the final question from §1 as follows: ‘Slow homeostatic mechanisms tune parameters of plasticity rules and neurons to enable efficient use of the available resources in networks’. For example, for the sake of efficiency, no neuron should *never* be active; no neuron should *always* be active; the number of neurons that respond to the exact same set of stimuli should stay limited.

## 10. Discussion

Taken together with the results from §§2–9, these insights suggest two distinct roles for negative feedback on different timescales. First, RCPs on short timescales stabilize Hebbian plasticity and make synapses onto the same neuron compete with each other. Heterosynaptic plasticity is likely to play a major role for these functionalities. Second, homeostatic mechanisms on slower timescales achieve fine-tuning of multiple network parameters. A slow shift of the threshold between LTD and LTP, the slow rescaling of all synaptic weights, or a slow regulation of neuronal parameters, are good candidates for these functionalities. Some of these slow mechanisms could be important only in setting up the network initially or after a strong external perturbation to the circuit. This view, however, raises an important question: Why do many modern plasticity models not include built-in RCPs, whereas classic models do?

### (a) Why are rapid compensatory processes missing in many spike-timing-dependent plasticity models?

Modern plasticity models try to capture a diversity of experimental data from rate-dependent [45], voltage-dependent [87] and spike-timing-dependent [68–70] plasticity experiments. One salient feature captured by most models [27,61,63,65,93] is the notion of a plasticity threshold which correlates with postsynaptic voltage, calcium concentration, postsynaptic firing rate, or other neuronal variables related to postsynaptic activation (figure 3*a*). Interestingly, most existing STDP models, although often explicitly fitted to data, are purely Hebbian and do not include the notion of RCPs. If such a rapid mechanisms exist—which is what we argue here—then how can it be that existing plasticity models without them can quantitatively capture the data from experiments?

There are presumably three main reasons for this. First, STDP experiments typically manipulate a single pathway, either by stimulating a presynaptic neuron or a bundle of presynaptic axons. Sometimes a designated control pathway (i.e. a second presynaptic neuron) is missing, or, if it is not missing, the effect size in the control pathway is considered as weak. However, from a theoretical perspective, we expect that heterosynaptic effects caused by stimulation of one presynaptic pathway are weak when measured at only one ‘control’ synapse; a weak change at individual synapses could still have a strong accumulated effect over thousands of synapses. Therefore, even weak heterosynaptic plasticity could act as a strong RCP [40,103,118].

Second, in an STDP experiment with 60 repetitions of pre-post-pairs, the total activation of the postsynaptic neuron is still in a reasonable regime. Therefore, it is unclear whether the ‘burst-detector’ for heterosynaptic plasticity would be triggered [40,103,118].

**Figure 8.**
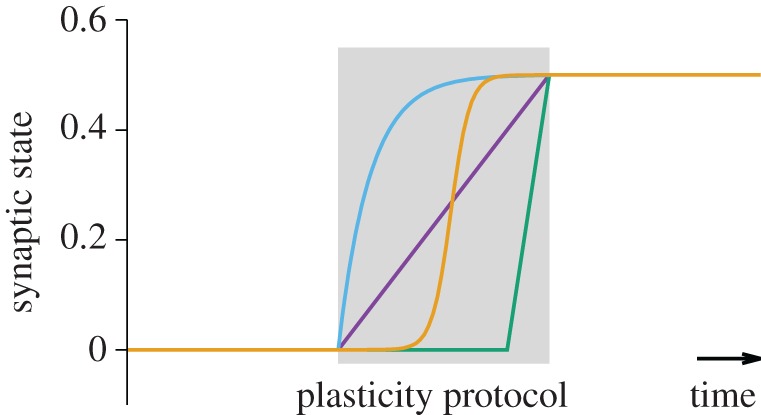
Hypothetical evolution of synaptic state variable as a function of time during a hypothetical LTP induction experiment. The linear ramp over the entire duration of the plasticity protocol is a common assumption underlying many plasticity protocols. However, since the synaptic state during plasticity induction is typically not observable, many time courses and thus plasticity modes are compatible with the data. Note, that most plasticity models will interpret the ‘synaptic state’ as the synaptic efficacy, whereas in experiments the synaptic efficacy may follow after a short delay.

Third, experiments typically rely on repeated pre- and postsynaptic activation. Moreover, during the induction protocol, synaptic efficacy changes are usually not observable. Plasticity models are thus fitted to pairs of initial and final synaptic strength. However, the unobserved intermediate synaptic dynamics during a standard LTP induction protocol could be qualitatively very different (figure 8), but are obscured in experiments by measurement artefacts as well as short-term plasticity riding on top of the induced Hebbian LTP. These differences in the dynamics contain the answers to questions such as: Is the final synaptic strength stable or would it increase further with additional pairings? Is there a threshold number of pairings that needs to be reached for an all or nothing effect?

Because the detailed internal dynamics of synapses during induction are not known, different plasticity models make different assumptions about the saturation of weights. Owing to the limited amount of experimental data, it is possible to construct a diversity of different models which are all consistent with the data. For instance, the Zenke *et al*. [103] model discussed in this paper is based on the triplet STDP model, and therefore consistent with existing STDP data, but it includes additional non-Hebbian RCPs. Although the presence of these added processes is important for network stability, their overall contribution to simulated STDP protocols is negligible. So, how can one verify or disprove the existence of RCPs experimentally?

### (b) How can we further constrain plasticity models by experiments?

There are multiple ways in which synaptic plasticity models could be constrained better through additional data. In the past, a large body of research has focused on homosynaptic associative plasticity, also called Hebbian plasticity, using pairing experiments with various protocols such as STDP. Here, we argue that heterosynaptic plasticity as well as transmitter-induced plasticity or similar stabilizing plasticity mechanisms are as important as Hebbian plasticity due to their crucial role for network stability.

*Heterosynaptic plasticity* and heterosynaptic effects mediated through metaplasticity [23,120,143] are promising candidates to stabilize Hebbian plasticity models against runaway LTP [26,40,103,118]. While heterosynaptic plasticity has been observed in various experiments [39,117], a conclusive picture and data-driven models are still scarce. Is it possible to measure the timescale, frequency dependence and weight dependence of neuron-wide heterosynaptic depression by manipulating the stimulation of the postsynaptic neuron? Does ‘pure’ heterosynaptic plasticity exist in the absence of presynaptic activity or is a slight activation of the presynaptic pathway always necessary to induce changes [120]? Another important question for the interpretation of heterosynaptic plasticity is whether it causes mostly synaptic depression similar to LTD or if it rather prevents or even resets early LTP through depotentiation at the unstimulated pathway [144]. Finally, the role of *heterosynaptic metaplasticity* [143] remains largely elusive.

*Transmitter-induced plasticity* is important in models and might be present in many experiments, even though it has not been reported as such. Here, we have interpreted transmitter-induced plasticity as a potentially weak form of LTP that is caused by presynaptic firing in the absence of postsynaptic activity. Why is this form of plasticity important? Suppose you have a network of neurons firing at low activity, so that any given neuron can be considered a weakly active postsynaptic neuron. As low activity typically induces LTD, many plastic network simulations have the tendency to fall silent. To compensate for this, theorists have either introduced lower bounds on synaptic weights or added weak LTP triggered by presynaptic activity [103,114]. How realistic are these assumptions?

Direct experimental evidence for such terms would, for instance, be the growth of synaptic efficacy during low activity ‘pre only’ stimulation. Such a term would manifest as a systematic positive drift of baseline in an experiment and could thus be easily interpreted as an unwanted instability [145,146]. From a theoretical standpoint, the importance of such a term—even if only weak—makes it an interesting target for future studies.

Finally, transmitter-induced plasticity could be replaced by a growth term without explicit presynaptic dependence. A plausible candidate for such a mechanism would for instance be spontaneous spine growth in the vicinity of a presynaptic axon. However, whether or not these rates would be on the correct timescale to compensate for LTD effects requires further theoretical investigation.

*Consolidation of synapses* is summarized in the present model by a reference weight 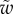 [54,103]. Simulations predict that synaptic consolidation renders synapses inert against heterosynaptic plasticity. Intuitively, the measured synaptic weights become ‘sticky’ and are always attracted back to their momentary stable state, i.e. weak or strong. This prediction requires future experimental clarification.

*The path towards saturation of synaptic weights* during a pairing experiment (figure 8) is vital to building better plasticity models. Virtually any information which helps theorists to constrain how the synaptic weight increases would be helpful. Importantly, this also includes any information about conditions (or experimental protocols) which do not induce plasticity, despite the fact that either the presynaptic or the postsynaptic neuron or both have been activated.

## 11. Conclusion

One of the most striking differences between plasticity models and experimental data concerns their timescales. Hebbian plasticity can be induced within seconds to minutes [45,68,69,87]. In simulated network models, a similarly fast form of Hebbian plasticity leads to runaway activity within seconds, unless Hebbian plasticity is complemented with RCPs. Here, ‘rapid’ means that these changes need to take effect after seconds or at most a few minutes [26]. This, however, is much faster than homeostatic plasticity observed in experiments. One of the most extensively studied forms of homeostasis in experiments is synaptic scaling [1] which proportionally scales synapses up or down when the network activity is too low or too high, respectively. However, even the fastest known forms of scaling take hours to days to cause measurable changes to synaptic weights (figure 1; [15,29,30]).

This apparent difference of timescales between RCPs required for stability in models and experimental results is a challenge for current theories [17,26,118,147]. To reconcile plasticity models and stability in networks of simulated neurons, we need to reconsider models of Hebbian plasticity and how they are fitted to data.

In most plasticity induction experiments, neither the time course of the manipulated synaptic state nor the precise changes of other synapses are observable during stimulation. Quantitative models of synaptic plasticity thus make minimal assumptions about these unobserved temporal dynamics and generally ignore heterosynaptic effects entirely. In other words, missing experimental data makes it possible to build different models which all capture the existing experimental data, but make different assumptions about the unobserved dynamics. Importantly, some of these models become intrinsically stable [10,57,118] or even multistable [17,103]. In most situations, these models can be interpreted as compound models consisting of Hebbian plasticity and forms of RCPs which only rely on quantities that are locally known to the synapse, i.e. the pre-postsynaptic activity as well as its own synaptic weight. Although such local forms of plasticity can solve the problem of stability at a neuronal level, in practice, most network models require additional fine-tuning of parameters to achieve plausible activity levels across a network of neurons. This role can be fulfilled by slow homeostatic mechanisms which act on timescales of hours or days, consistent with experimental data on homeostatic plasticity.

In summary, several theoretical arguments suggest that Hebbian plasticity is intrinsically stabilized on short timescales by RCPs, likely to be implemented as heterosynaptic plasticity, or network-wide negative feedback mechanisms. Slow forms of homeostatic plasticity, on the other hand, set the stage for stable learning. This hypothesis will now have to stand the test of time. It will thus be an important challenge for the coming years to go beyond homosynaptic Hebbian plasticity and to gain a more complete understanding of its interactions with a diversity of compensatory processes across timescales.

## Supplementary Material

Supplementary Text

## Endnotes

1 As an aside, we note that the term ‘heterosynaptic plasticity’ is sometimes also used for synaptic changes that are visible at the connection from a presynaptic neuron *j* to a postsynaptic neuron *i*, but induced by the activation of a third, typically modulatory neuron [78]. However, in this paper we do not consider this possibility.

2 Strictly speaking, the notion of a ‘timescale’ is meaningless for a nonlinear differential equation like equation (3.3), it is only defined for linear dynamical systems. The notion can be rescued, however, in the vicinity of a fixed point around which the system can be linearized. We refer the interested reader to the electronic supplementary material where we provide additional information on this important, but technical issue.
