# Supplementary material for: Hebbian plasticity requires compensatory processes on multiple timescales
Source: Philos Trans R Soc Lond B Biol Sci. 2017 Mar 5;372(1715):20160259. doi: 10.1098/rstb.2016.0259 (PMC5247595; doi:10.1098/rstb.2016.0259)
Supplement: Supplementary Text [file rstb20160259supp1.pdf]

Electronic supplementary material: Hebbian plasticity requires compensatory processes on multiple timescales

Friedemann Zenke[1,*] and Wulfram Gerstner[2]

1) Dept. of Applied Physics, Stanford University, Stanford, CA 94305, USA
orcid.org/0000-0003-1883-644X
2) Brain Mind Institute, School of Life Sciences and School of Computer and Communication Sciences, Ecole Polytechnique Fédérale de Lausanne, CH-1015 Lausanne EPFL, Switzerland
orcid.org/0000-0002-4344-2189

Published as: Zenke F, Gerstner W. 2017 Hebbian plasticity requires compensatory processes on multiple timescales. Phil. Trans. R. Soc. B 20160259.
http://dx.doi.org/10.1098/rstb.2016.0259

*Author for correspondence (fzenke@stanford.edu)

S1 Mathematical digression: The notion of a timescale

To formally introduce the notion of a timescale, we consider a linear system defined by the differential equation

$$\tau \frac{dy}{dt} = -(y - \xi) \tag{S1}$$

where ξ and τ are two fixed positive parameters. The solution $y(t)$ of the differential equation relaxes exponentially to the value $y = \xi$, on the timescale τ. Practically speaking that means that no matter how we initialize $y(t)$ at time $t = 0$, if we wait for a time 3τ, the difference $y(t) - \xi$ will have decreased by 95%. The exponential evolution is typical for a linear system. Moreover, the equation has a nice scaling behavior: If we know the solution for a parameter setting $\tau = 1$, we can get the solution for the parameter setting $\tau = 5$ by multiplying all times by a factor of five. This is the reason why we speak of τ as the timescale of the system.

The fact that a constant value of $y(t) = \xi$ for all t is a solution means that ξ is a fixed point of the system. To check this, remember that the derivative of a constant is zero (so that the left-hand side of Eq. (S1) vanishes). And for $y = \xi$ the right-hand side is obviously zero, too. Moreover, this fixed point is stable because from any initial condition, $y(t)$ will converge towards ξ.

Let us now consider the case in which τ is negative. In this case, $y(t) = \xi$ is still a fixed point, but if we start with a value $y(t) > \xi$, then $y(t)$ will explode exponentially fast to large positive values while for an initial value $y(t) < \xi$ it will explode exponentially fast to large negative values. The timescale of explosion is again given by τ.

However, our intuition for timescales breaks down for nonlinear systems. Therefore, when we consider a nonlinear system $\tau \frac{dy}{dt} = f(y)$, it does not suffice for τ to be large to be able to talk about a slow or fast timescale. We have to be specific about the behavior of f in the regime that we are interested in. The mathematical trick to do this is to look for a fixed point of the equation, that is a value of y with $f(y) = 0$. Suppose $y = y_0$ is a fixed point. We then study the derivative df/dy at y_0. Let us denote this derivative by f'. In the neighborhood of y_0 (and only there!) the nonlinear equation is well approximated by a linear equation $\tau dy/dt = (y - y_0)\, f'$. Division by f' brings f' to the other side of the equation and enables us to identify the *effective timescale* $\tilde{\tau} = -\tau/f'$. If you are in doubt, compare your result with equation (S1).

Another example of a linear system is a low-pass filter. Suppose we pass our variable $y(t)$ through a low-pass filter with time constant τ_d to yield

$$\bar{y}(t) = \int_{-\infty}^{t} \exp\left[-\frac{(t - t')}{\tau_d}\right] y(t')\, dt' \,. \tag{S2}$$

By taking the derivative on both sides of Eq. (S2), we find that the low-pass filter $\bar{y}$ is the solution of a differential equation $d\bar{y}(t)/dt = -\bar{y}(t)/\tau_d + y(t)$. This equation is similar to equation (S1), if we replace the constant target value ξ by a time-dependent target $y(t)/\tau_d$.

S2 The induction timescale of Hebbian plasticity

Because both learning rules and neurons are typically nonlinear, the mathematical definition of a timescale for Hebbian plasticity requires the existence of a fixed point. The reason is that for a nonlinear system we can define a timescale only in the vicinity of a fixed point, as we have seen above.

Even if a plasticity rule in isolation looks linear, the combination of plasticity with the neuronal dynamics typically makes the network as a whole nonlinear. Consider for instance a single neuron i that is driven by N inputs arriving at synapses w_{ij} for $1 \leq j \leq N$. In a rate model, the state of the postsynaptic neuron is characterized by its firing rate $y_i = g\left(\sum_{j=1}^{N} w_{ij} x_j\right)$ where x_j is the firing rate of the presynaptic neuron with index j. The function g denotes the frequency-current relation of a single neuron and we assume that it is monotonically increasing, i.e., if we increase the input, the firing rate increases as well. In the theoretical literature g is sometimes called the gain function of the neuron, hence our choice of letter g.

Let us first study a simple Hebbian learning rule

$$\frac{dw_{ij}}{dt} = \eta \, y_i \, x_j \, . \tag{S3}$$

Note that this is a special instance within the general framework of Eqs. (1,2) in the main manuscript. To see this, consider a Hebbian term $H(\mathrm{post}_i, \mathrm{pre}_j) = H(y_i, x_j) = y_i \, x_j$, and $a_1(w_{ij}) = \eta$ and set all other terms in Eq. (2) to zero. This learning rule is linear in x_j and linear in y_i. However, if we insert $y_i = g\left(\sum_j w_{ij} x_j\right)$ into the learning rule, the learning dynamics become nonlinear in x_j. Nonlinearity implies that we cannot define a timescale of plasticity, unless we find a fixed point where dw_{ij}/dt vanishes.

Are there fixed points of the dynamics? There is a fixed point if the postsynaptic or the presynaptic rate is zero. However, if the neuron is embedded in a network, it is reasonable to assume that several presynaptic neurons including the presynaptic neuron j are active. Unless all weights w_{ij} are zero, the postsynaptic neuron is therefore also active, and the weight w_{ij} increases.

The argument can be formalized to show that $w_{ij} = 0$ is an unstable fixed point. If we increase w_{ij} by just a little, the output y_i also increases which increases w_{ij} even further, which closes the positive feedback loop. More generally, models of Hebbian plasticity that are useful for memory formation all have an unstable fixed point. One important role of Rapid Compensatory Processes (RCPs) in network models with Hebbian plasticity is therefore to create (additional) stable fixed points for the learning dynamics as we will see later on in this section.

The simple example above only has a trivial fixed point at zero activity (zero weights). Moreover, it is lacking the notion of long-term depression (LTD). Plausible plasticity models have additional stationary points defined by the plasticity threshold between long-term potentiation (LTP) and LTD (Fig. 2a). Inspired by experimental data [1–3], the transition from LTP to LTD depends in

models on the state of the postsynaptic neuron, e.g., its membrane potential, calcium level, inter spike interval [4–8]. As a paradigmatic example, which stands for a plethora of different plasticity rules with a postsynaptic threshold, we consider the following rate-based nonlinear Hebbian rule

$$\frac{dw_{ij}}{dt} = \eta\, x_j y_i \, (y_i - \theta) \tag{S4}$$

with a positive constant η. Whenever the postsynaptic firing rate y_i equals the value of θ, the weight w_{ij} does not change. Thus this learning rule has a fixed point at the threshold $y_i = \theta$. For y_i larger than θ the synaptic weights increase which corresponds to the induction of LTP in the model. For y_i smaller than θ the synaptic weights decrease and LTD is induced.

Let us now embed this learning rule in a network. For the sake of simplicity we assume the gain function to be linear, $g\left(\sum_j w_{ij} x_j\right) = \sum_j w_{ij} x_j$. Moreover, we assume that (i) all weights w_{ij} have the same value $w_{ij} = w$ and (ii) all N neurons in the network fire with the same firing rate $x_j = 1/N$, so that $y = w$. Inserting these assumptions into Eq. (S4) yields

$$\frac{dw}{dt} = \eta\, w \,(w - \theta) \tag{S5}$$

which characterizes the weight change induced by synaptic plasticity. We now linearize Expression (S5) at the stationary point $w = \theta$:

$$\frac{dw}{dt} \approx \eta\, \theta \;(w - \theta)$$

This is a linear differential equation with a solution $w(t)$ that, for $w(t) > \theta$, explodes exponentially fast on the timescale $\tau = \frac{1}{\eta\theta}$ (cf. discussion of Eq. (S1)). Thus, in this example, $w = \theta$ is an unstable fixed point and the linearization procedure has enabled us to identify the timescale of plasticity induction. In practical implementations of a plasticity model, the exponential growth of the synaptic weights w_{ij} would stop when they obtain their maximal value $w^{\max}$. However, if all synapses onto a postsynaptic neuron, or even all synapses in a neural network sit at their upper bound, the network cannot function as a memory.

Note that the occurrence of the parameter θ in the timescale τ is a hallmark of the nonlinearity of the full system. Just like in the well-known Hodgkin-Huxley model where the timescale of the activation and inactivation variable depends on the voltage, the effective timescale τ of a learning rule will depend on multiple factors such as the presynaptic activity, the slope of the gain function, or the threshold θ between LTD and LTP.

Even though τ in the example above is not the same as the induction timescale of long-term plasticity, in experiments in which only a single presynaptic pathway is stimulated, the two are related. With our formalism we can also account for the strength of the recurrent feedback that is received by a neuron embedded into a network. Just repeat the above analysis under the assumption

that the presynaptic and postsynaptic neurons are mutually connected, of the same type, and fire at the same rate [9]. Whatever you consider as a reasonable scenario, the timescale τ characterizes how quickly a neuron or an entire recurrent network can generate positive feedback and is able to "run away".

Instead of writing down a differential equation for the weights w, as in Eq. (S5), we could have written the system in terms of y, too. A formulation in terms of the firing rates y highlights the fact that we have to think about run-away effects of synapses as being linked to run-away effects of neuronal activity. Note further, that in realistic neuronal networks the presynaptic activity fluctuates and is different between one neuron and the next. Fluctuations give rise to a covariance matrix $C = \frac{1}{n}\sum_{t=1}^{n} x_i(t)x_j(t)$ which may look complicated at a first glance. However, due to the symmetry of C, there always exists a basis in which C is diagonal. When working in this basis, the plasticity equations decouple and take the shape of Eq. (S5) with different values of η.

This analytical formalism is quite general [9] (see Yger and Gilson [10] for a review) and can be applied to spike-timing-dependent plasticity (STDP), spiking neurons and spiking neural networks. However, because such systems are often stochastic and usually high-dimensional it can become intractable to explicitly compute the state of each neuron x_i and each synapse w_{ij}. However, it is often possible to consider instead the average change across all synapses $\left\langle \frac{dw_{ij}}{dt} \right\rangle$ and activities $\left\langle \frac{dy_i}{dt} \right\rangle$ respectively. Under fairly general conditions these averages take the functional form of the simple rate models above which are low-dimensional and analytically tractable. Despite this enormous dimensionality reduction from a large spiking neural network with a quantitative STDP model, these mean field models give surprisingly accurate quantitative predictions about bifurcation parameters, such as τ_{crit}, at which instability occurs [9].

References

[1] A. Artola, S. Bröcher, and W. Singer. Different voltage-dependent thresholds for inducing long-term depression and long-term potentiation in slices of rat visual cortex. *Nature*, 347(6288): 69–72, September 1990. doi: 10.1038/347069a0.

[2] S M Dudek and M F Bear. Homosynaptic Long-Term Depression in Area CA1 of Hippocampus and Effects of N-Methyl-D-Aspartate Receptor Blockade. *Proc Natl Acad Sci U S A*, 89(10): 4363–4367, May 1992.

[3] Per Jesper Sjöström, Gina G Turrigiano, and Sacha B Nelson. Rate, Timing, and Cooperativity Jointly Determine Cortical Synaptic Plasticity. *Neuron*, 32(6):1149–1164, December 2001. doi: 10.1016/S0896-6273(01)00542-6.

[4] A Artola and W Singer. Long-term depression of excitatory synaptic transmission and its relationship to long-term potentiation. *Trends Neurosci*, 16(11):480–487, November 1993.

[5] Harel Z. Shouval, Mark F. Bear, and Leon N. Cooper. A Unified Model of NMDA Receptor-Dependent Bidirectional Synaptic Plasticity. *Proc Natl Acad Sci U S A*, 99(16):10831–10836, August 2002. doi: 10.1073/pnas.152343099.

[6] Jean-Pascal Pfister and Wulfram Gerstner. Triplets of Spikes in a Model of Spike Timing-Dependent Plasticity. *J Neurosci*, 26(38):9673–9682, September 2006. doi: 10.1523/JNEUROSCI.1425-06.2006.

[7] Claudia Clopath and Wulfram Gerstner. Voltage and spike timing interact in STDP – a unified model. *Front Synaptic Neurosci*, 2:25, 2010. doi: 10.3389/fnsyn.2010.00025.

[8] Michael Graupner and Nicolas Brunel. Calcium-based plasticity model explains sensitivity of synaptic changes to spike pattern, rate, and dendritic location. *Proc Natl Acad Sci U S A*, 109 (10):3991–3996, March 2012. doi: 10.1073/pnas.1109359109.

[9] Friedemann Zenke, Guillaume Hennequin, and Wulfram Gerstner. Synaptic Plasticity in Neural Networks Needs Homeostasis with a Fast Rate Detector. *PLoS Comput Biol*, 9(11):e1003330, November 2013. doi: 10.1371/journal.pcbi.1003330.

[10] Pierre Yger and Matthieu Gilson. Models of Metaplasticity: A Review of Concepts. *Front. Comput. Neurosci*, page 138, 2015. doi: 10.3389/fncom.2015.00138.
